# Supplementary material for: High Levels of the Cleaved Form of Galectin-9 and Osteopontin in the Plasma Are Associated with Inflammatory Markers That Reflect the Severity of COVID-19 Pneumonia
Source: Int J Mol Sci. 2021 May 7;22(9):4978. doi: 10.3390/ijms22094978 (PMC8125627; doi:10.3390/ijms22094978)
Supplement: Supplementary file 1 [file ijms-22-04978-s001.zip › Supplementary file 507r (2).pdf]

Table S1. Statistical data of the ROC analysis of the CV, CP, and ID groups versus the HC group**.**

	Biomarker	Threshold	Specificity	Sensitivity	Accuracy	NPV	PPV	Youden
HC vs CV	Tr-Gal9	635	0.97	0.70	0.85	0.81	0.94	1.66
	FL-Gal9	105	0.77	0.70	0.74	0.77	0.70	1.46
	Ud-OPN	20.0	0.90	1.00	0.94	1.00	0.88	1.90
	FL-OPN	162	0.93	1.00	0.96	1.00	0.92	1.93
HC vs CP	Tr-Gal9	654	0.97	1.00	0.98	1.00	0.96	1.97
	FL-Gal9	237	0.93	0.88	0.91	0.90	0.92	1.81
	Ud-OPN	40.4	1.00	0.96	0.98	0.97	1.00	1.96
	FL-OPN	206	0.97	0.96	0.96	0.97	0.96	1.93
HC vs ID	Tr-Gal9	648	0.97	0.93	0.95	0.97	0.93	1.90
	FL-Gal9	76.1	0.63	0.92	0.71	0.95	0.50	1.55
	Ud-OPN	60.3	1.00	1.00	1.00	1.00	1.00	2.00
	FL-OPN	162	0.93	1.00	0.95	1.00	0.88	1.93

Table S2. Statistical data of the ROC analysis of the CP group versus the CV group.

	Biomarker	Threshold	Specificity	Sensitivity	Accuracy	NPV	PPV	Youden
CV vs CP	Tr-Gal9	1250	0.78	0.88	0.83	0.86	0.81	1.66
	FL-Gal9	222	0.65	0.92	0.79	0.88	0.74	1.57
	Ud-OPN	52.7	0.78	0.72	0.75	0.72	0.78	1.50
	FL-OPN	558	1.0	0.4	0.69	0.61	1.00	1.40
	CRP	0.53	0.96	0.88	0.92	0.88	0.96	1.84
	sIl-2R	724	0.95	0.50	0.72	0.64	0.92	1.45
	Ferritin	293	0.91	0.75	0.83	0.77	0.90	1.66
	D-dimer	0.57	0.55	0.88	0.72	0.80	0.69	1.43
	B2M	562	0.95	0.50	0.71	0.63	0.92	1.45
	SpO_2	96.5	0.78	0.64	0.71	0.67	0.76	1.42
	S/F	460	0.78	0.64	0.71	0.67	0.76	1.42
	Creatinine	1.09	1.00	0.32	0.65	0.58	1.00	1.32
	Lymphocytes	1246	0.77	0.63	0.70	0.65	0.75	1.40

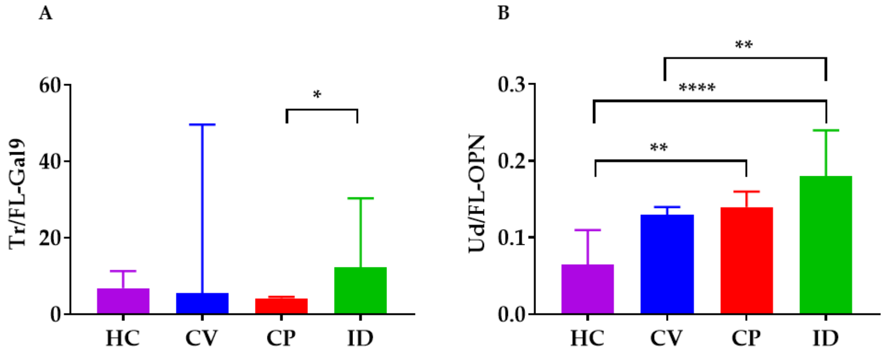

Supplementary figure S1.

Ratios of Tr-Gal9/FL-Gal9 (A) and Ud-OPN/FL-OPN (B) in the CV, CP, ID, and HC groups. HC; healthy control, CV: COVID-19 infection with mild clinical symptoms, CP: COVID-19 associated with pneumonia, ID; Infectious diseases FL-Gal9; Full- length- Gal-9, Tr-Gal9; truncated Gal-9., FL-OPN; full-length OPN, Ud-OPN; undefined OPN, ****; P<0.0001, ***; p<0.001, **; p<0.01, *; p<0.05.

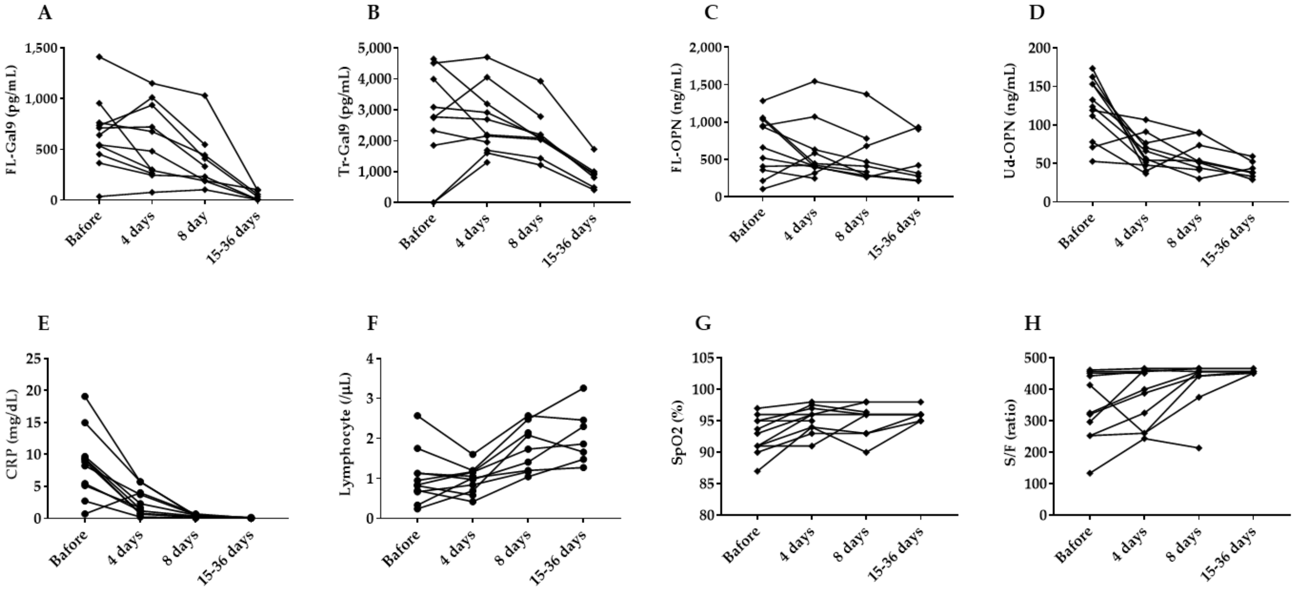

Supplementary figure S2. Time course of inflammatory, coagulation, kidney and respiratory indicators during TCZ therapy in each patient. FL-Gal9 (A), Tr-Gal9 (B), FL-OPN (C), Ud-OPN (D), CRP (E), Lymphocyte numbers (F), SpO2 (G), S/F ratio (H).

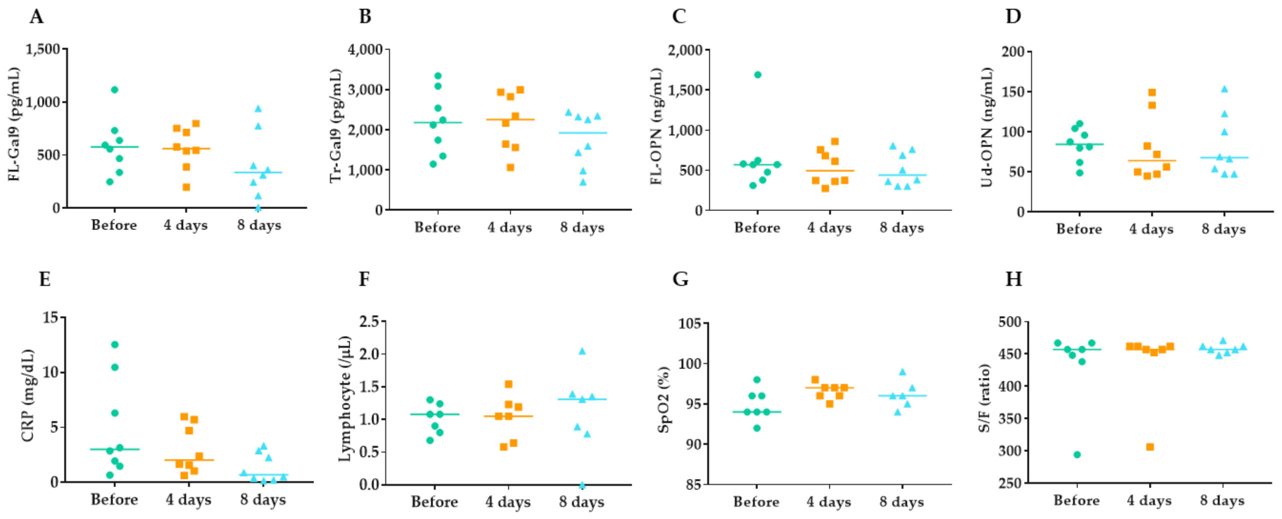

Supplementary figure S3. Time course of inflammatory, coagulation, kidney and respiratory indicators during therapy without TCZ. FL-Gal9 (A), Tr-Gal9 (B), FL-OPN (C), Ud-OPN (D), CRP (E), Lymphocyte (F), SpO2 (G), S/F ratio (H).
